# Reduced lymphocyte count as an early marker for predicting infected pancreatic necrosis

**DOI:** 10.1186/s12876-015-0375-2

**Published:** 2015-10-26

**Authors:** Xiao Shen, Jing Sun, Lu Ke, Lei Zou, Baiqiang Li, Zhihui Tong, Weiqin Li, Ning Li, Jieshou Li

**Affiliations:** 1 Surgical Intensive Care Unit (SICU), Department of General Surgery, Jinling Hospital, Medical School of Nanjing University, No. 305 Zhongshan East Road, Nanjing, 210002 Jiangsu Province China; 2 Department of General Surgery, Jinling Hospital, Medical School of Nanjing University, No. 305 Zhongshan East Road, Nanjing, 210002 Jiangsu Province China

**Keywords:** Acute pancreatitis, Infected pancreatic necrosis, Lymphocyte count, Immunosuppression

## Abstract

**Background:**

Early occurrence of immunosuppression is a risk factor for infected pancreatic necrosis (IPN) in the patients with acute pancreatitis (AP). However, current measures for the immune systems are too cumbersome and not widely available. Significantly decreased lymphocyte count has been shown in patients with severe but not mild type of AP. Whereas, the correlation between the absolute lymphocyte count and IPN is still unknown. We conduct this study to reveal the exact relationship between early lymphocyte count and the development of IPN in the population of AP patients.

**Methods:**

One hundred and fifty-three patients with acute pancreatitis admitted to Jinling Hospital during the period of January 2012 to July 2014 were included in this retrospective study. The absolute lymphocyte count and other relevant parameters were measured on admission. The diagnosis of IPN was based on the definition of the revised Atlanta classification.

**Results:**

Patients were divided into two groups according to the presence of IPN. Thirty patients developed infected necrotizing pancreatitis during the disease course. The absolute lymphocyte count in patients with IPN was significantly lower on admission (0.62 × 10^9^/L, interquartile range [IQR]: 0.46–0.87 × 10^9^/L vs. 0.91 × 10^9^/L, IQR: 0.72–1.27 × 10^9^/L, *p* < 0.001) and throughout the whole clinical course than those without IPN. Logistic regression indicated that reduced lymphocyte count was an independent risk factor for IPN. The optimal cut-offs from ROC curve was 0.66 × 10^9^/L giving sensitivity of 83.7 % and specificity of 66.7 %.

**Conclusions:**

Reduced lymphocyte count within 48 h of AP onset is significantly and independently associated with the development of IPN.

## Background

Acute pancreatitis (AP) is a sudden inflammation of the pancreas with a mortality rate of 6–10 % [1]. In the past, the Atlanta classification was commonly used to grade the severity of AP, briefly, mild and severe AP. Patients with severe acute pancreatitis (SAP) are usually associated with multiple organ dysfunction (MODS) and poor prognosis. About 5–10 % of the AP patients would develop necrosis of the pancreatic or peripancreatic tissue [2] and the necrotic tissue can remain sterile or be infected, becoming infected pancreatic necrosis (IPN). IPN is known to be an independent risk factor for ultimate mortality [3] and always develops during the second or third week after the onset of the disease [4, 5]. It is recently reported that more than 80 % of the mortality occurs at the late stage as a result of infection [6]. Thus, it is of great importance to distinguish those patients with higher risk for IPN at the initial stage of the disease and make preventive intervention.

Recently, more and more studies have shown that immunosuppression is a key pathogenesis of SAP. Early alterations of the immune system comprising decreased activation of T lymphocyte [7] and down regulation of human leukocyte antigen (HLA) DR [8] may cause IPN, leading to multiple organ failure (MOF) and high mortality. In this way, these immunological indexes might be useful predictors for the prognosis of AP patients. However, due to the complexity of the measurement methods, the abovementioned indexes are not monitored routinely in the clinical work. Recently, the prognostic value of the neutrophil-lymphocyte ratio (NLR) had also been evaluated, but turned out with controversial results [9]. The absolute lymphocyte count was also assessed as an important part of the immune system. In 1985, Christophi et al. first declared the absolute lymphocyte count had a prognostic significance in the severity of acute pancreatitis [10]. Whereas, no further studies regarding the role of the absolute lymphocyte count as an independent factor for disease severity or mortality in AP patients were carried out after that [11].

Our investigation was the first study to compare the early alteration of the absolute lymphocyte count in the peripheral blood of AP patients with and without IPN. The aim of this study was to determine whether the lymphocyte count was a strong predictor for IPN in patients with acute pancreatitis and how strongly the absolute lymphocyte count was associated with the prognosis.

## Methods

### Patients

This was a retrospective observational study conducted in the Department of General Surgery, Jinling Hospital, China. The data collection for this study was approved by the Institutional Review Board of our hospital. One hundred fifty-three patients with a diagnosis of acute pancreatitis consecutively treated in our center during January 2012 to July 2014 were included for potential analysis. The diagnostic criteria of AP were according to two of the following three clinical features [2]: upper abdominal pain, significantly increased serum levels of lipase (or amylase) activity and imaging findings consistent with acute pancreatitis. The inclusion criteria were AP patients aged 18 years or older who admitted to our center within 48 h after the onset of the disease and received systemic laboratory evaluations on admission. Patients during pregnancy, with a history of cancer or bone marrow diseases or a medical history of immunosuppressive agents were excluded from this study.

### Data collection

All the patients with AP were evaluated for blood routine and biochemical tests at arrival in the central laboratory of our hospital. Hemoglobin, hematocrit, platelet, C-reactive protein (CRP), white blood cells as well as the absolute neutrophil and lymphocyte counts were obtained from an automatic blood cell analyzer (CELL-DYN3700, Chicago, Abbott). NLR was calculated using the values of the absolute neutrophil and lymphocyte counts. Serum levels of albumin, amylase and lipase were detected using an Aeroset (Hitachi 7060 Automatic Biochemical Analyzer, Tokyo, Japan). HLA-DR and T lymphocyte subsets were also assayed in our central laboratory in 30 patients (24 in non-IPN group and 6 in IPN group) by the direct fluorescence method for the whole blood using flow cytometer in a flow cytometer (FACS-Calibur, Becton Dickinson, San Jose, Calif., USA) and double straining (FITC/PE) monoclonal antibodies (Marseilles, France). HLA-DR expression was measured in the monocyte population and T lymphocyte subsets were measured in the lymphocytes population. Analysis of the data was performed by CellQuest software. Demographic variables, possible etiology of acute pancreatitis were reviewed and recorded by two independent physicians.

Baseline characteristics, including age, gender, body mass index, Acute Physiology and Chronic Health Enquiry II (APACHE II) score and computed tomography (CT) severity index, were also collected and recorded.

### Definition

The diagnosis of IPN was according to the imaging findings and/or bacterial culture result: either the presence of extraluminal gas in the pancreatic and/or peripancreatic tissues on contrast-enhanced computed tomography (CECT) or positive bacterial culture of aspiration and drainage content of pancreatic and/or peripancreatic tissues could confirm the diagnosis [2]. Lymphocytopenia was defined as the absolute lymphocyte count below 0.8 × 10^9^/L. Organ dysfunction was evaluated in three organ systems (respiratory, renal and cardiovascular) within 24 h after admission and the definition of organ dysfunction was based on the modified Marshall scoring system, defining as a score of 2 or more [12]. The definition of local complications, including portal vein thrombosis, intra-abdominal hypertension and hemorrhage, deep vein thrombosis (DVT) and gastrointestinal fistula, judged by two independent physicians, was according to the recently revised Atlanta criteria [2]. Disease severity of AP was assessed based on the presence of sterile/infected pancreatic necrosis and transient/persistent organ failure, namely, mild, moderate, severe and critical AP [13].

### Statistic analysis

Continuous variables in the data were presented as medians plus interquartile range (IQR) and categorical variables were presented as absolute numbers and percentage. Mann–Whitney *U* test was used in continuous variables and Chi-square test was used to analyze categorical variables for group comparisons. Logistic regression was constructed to evaluate the relationship between the relevant parameters and secondary infection. Multivariate logistic regression only involved in the variables that showed statistic significance in univariate analysis. Further receiver operating characteristic (ROC) curve was displayed for accuracy assessment. Statistical analyses were performed using SPSS (version 22.0) statistical software (IBM Analytics, Armonk, NY). A probability (p value) of <0.05 was considered statistically significant.

## Results

**Fig. 1:**
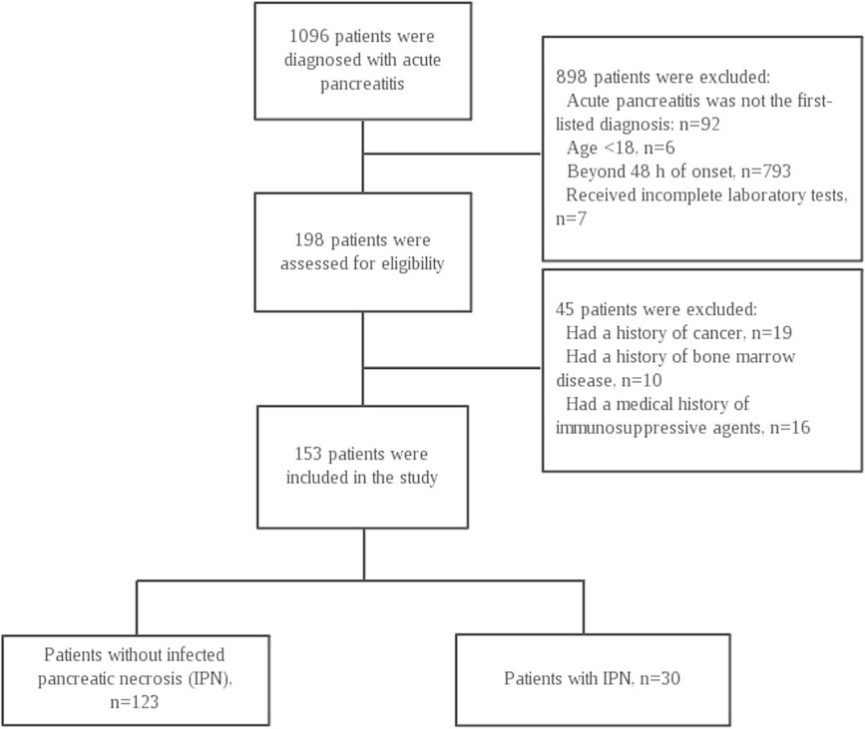
Screening and grouping of the study patients

**Fig. 2:**
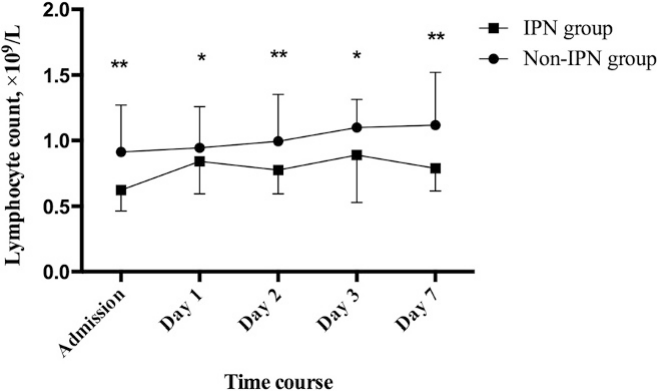
Change of the absolute lymphocyte count during the disease course of acute pancreatitis in the patients of different groups. Values were presented with median ± interquartile range (IQR); IPN: infected pancreatic necrosis. *p < 0.05 for IPN vs. non-IPN group, **p < 0.001 for IPN vs. non-IPN group

**Fig. 3:**
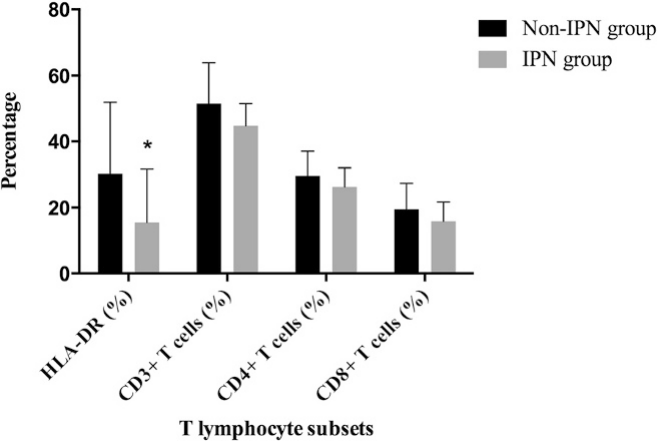
Change of Human leukocyte antigen (HLA)-DR and T lymphocyte subsets in patients of different groups. Values were presented with median ± IQR; IPN: infected pancreatic necrosis. *p < 0.05 for IPN vs. non-IPN group, **p < 0.001 for IPN vs. non-IPN group

1096 patients were initially screened for the study and eventually 153 patients were enrolled for analysis (Fig. 1). Patients were divided into two groups according to the presence of IPN, namely, IPN (*n* = 30) and non-IPN (*n* = 123) group. Except for the acute physiology and chronic health enquiry II (APACHE II) score, the baseline characteristics showed no significant differences between the two groups (Table 1). Also, no significant difference was seen in the distribution of etiology. Lymphocytopenia developed in 42 (34.1 %) patients in non-IPN group and 19 (63.3 %) patients in IPN group (*p* = 0.006). The median absolute lymphocyte count in peripheral blood of the patients with IPN was significantly lower than those patients without IPN on admission (0.62 × 10^9^/L, interquartile range [IQR]: 0.46–0.87 × 10^9^/L vs. 0.91 × 10^9^/L, IQR: 0.72–1.27 × 10^9^/L, p < 0.001) and also in the following days (Fig. 2). Consistent with the previous studies, the median value of NLR was significantly higher in the patients with IPN compared to those without (15.5, IQR: 12.0–23.8 vs. 10.9, IQR: 7.7–18.2, *p* = 0.002), indicating immunosuppression occurred at the very onset of AP in the patients with IPN. As for other immunological indexes, we also measured the expression of HLA-DR on peripheral monocytes and T lymphocyte subsets on peripheral lymphocytes. Down-regulated expression of HLA-DR (15.5 %, IQR: 12.9–31.7 % vs. 30.3 %, IQR: 22.4–51.9 %, *p* = 0.026) and decreased proportion of mature T lymphocytes (44.8 %, IQR: 31.4–51.5 % vs. 51.5 %, IQR: 40.4–63.9 %, *p* = 0.203) in the patients with IPN also confirmed the presence of immunosuppression (Fig. 3). As a result of immunosuppression, patients who developed IPN suffered higher mortality and longer hospital durations.

**Table 1:** Baseline characteristics and clinical features of the AP patients with or without IPN

Variable	Non-IPN group (*n* = 123)	IPN group (*n* = 30)	*p* Value
Age, years	45 (36, 56)	47 (38, 57)	0.529
Gender, male/female	79/44	21/9	0.068
BMI	26.1 (23.3, 28.8)	27.3 (23.0, 28.4)	0.155
APACHE II score	13 (12, 15)	20 (14, 29)	<0.001
CT severity index	3 (3, 6)	8 (6, 8.5)	<0.001
Laboratory data			
Hemoglobin, g/L	135 (116, 150)	126 (101, 149)	0.247
Hematocrit	0.40 (0.34, 0.45)	0.36 (0.29, 0.44)	0.211
Platelet, ×10^9^/L	158 (117, 204)	116 (81, 166)	0.002
CRP, mg/L	161.0 (71.8, 208.8)	202.7 (158.7, 251.5)	0.001
WBC, ×10^9^/L	13.0 (9.6, 17.3)	11.8 (8.7, 14.6)	0.152
Lymphocyte count, ×10^9^/L	0.91 (0.72, 1.27)	0.62 (0.46, 0.87)	<0.001
NLR	10.9 (7.7, 18.2)	15.5 (12.0, 23.8)	0.002
Albumin, g/L	35.6 (34.0, 38.2)	34.4 (31.6, 36.2)	0.010
Amylase, U/L	276 (138, 544)	270 (183, 934)	0.129
Lipase, U/L	1095 (457, 1916)	972 (579, 1814)	0.963
Aetiology of acute pancreatitis, no. (%)			
Gallstone	59 (48.0)	14 (46.7)	1.000
Hypertriglyceridemia	49 (39.8)	15 (50.0)	0.409
Alcohol	11 (8.9)	1 (3.3)	0.462
Post-ERCP	2 (1.6)	0 (0)	1.000
Other	2 (1.6)	0 (0)	1.000

*AP* acute pancreatitis, *IPN* infected pancreatic necrosis, *BMI* Body Mass Index, *APACHE II score* Acute Physiology and Chronic Health Enquiry II score, *CT* computed tomography, *CRP* C-reactive protein, *WBC* white blood cells, *NLR* Neutrophil-lymphocyte ratio, *ERCP* Endoscopic Retrograde Cholangiopancreatography

Table 2 represented the comparison of complications and outcome between the two groups. The incidence of organ dysfunction was 30.9 % (38/123) in the patients of non-IPN group and 86.7 % (26/30) in those of IPN group (*p* < 0.001). Acute respiratory distress syndrome (ARDS) and acute kidney injury (AKI) were two most common organ dysfunctions in both groups, followed by shock, mainly septic shock. Higher percentage of organ dysfunctions in the patients with IPN led to increased need for mechanical ventilation and continuous renal replacement therapy (CRRT). Intra-abdominal hemorrhage and hypertension were the most common complications in both two groups, followed by fistula. Four patients in the non-IPN group died during hospitalization: MOF for two patients, pulmonary embolism and unexplained cardiac arrest for the remaining two patients, respectively. The hospital mortality was much higher in the patients with IPN, above 40 %. All patients but one died of MOF.

**Table 2:** Complications and outcomes of the AP patients with or without IPN

	Non-IPN group (*n* = 123)	IPN group (*n* = 30)	*p* Value
Severity of AP, no. (%)			<0.001
Mild	59 (48.0)	0 (0)	
Moderate	26 (21.1)	0 (0)	
Severe	38 (30.9)	4 (13.3)	
Critical	0 (0)	26 (86.7)	
Organ dysfunction, no. (%)			
Respiratory	34 (27.6)	19 (63.3)	<0.001
Renal	20 (16.3)	22 (73.3)	<0.001
Cardiovascular	5 (4.1)	12 (40.0)	<0.001
Mechanical ventilation, no. (%)	21 (17.1)	18 (60.0)	<0.001
CRRT, no. (%)	20 (16.3)	19 (63.3)	<0.001
Complication, no. (%)			
Pancreatic pseudocyst	8 (6.5)	1 (3.3)	1.000
Invasive fungal infection	0 (0)	2 (6.7)	0.037
Intra-abdominal hemorrhage	1 (0.8)	9 (30.0)	<0.001
Deep vein thrombosis	5 (4.1)	2 (6.7)	0.624
Portal thrombosis	1 (0.8)	1 (3.3)	0.355
IAH	6 (4.9)	8 (26.7)	0.001
Encephalopathy	1 (0.8)	0 (0)	1.000
Fistula	0 (0)	5 (16.7)	<0.001
Hospital stay, days	10 (6, 14)	30 (18, 54)	<0.001
ICU stay, days	6 (3, 9)	17 (9, 48)	<0.001
Mortality rate, no. (%)	4 (3.3)	13 (43.3)	<0.001

*IPN* infected pancreatic necrosis, *AP* acute pancreatitis, *CRRT* continuous renal replacement therapy, *IAH* intra-abdominal hypertension, *ICU* intensive care unit

**Fig. 4:**
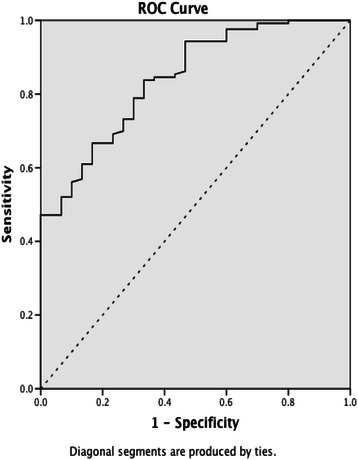
Receiver operating characteristic (ROC) curve for the absolute lymphocyte count in predicting infected pancreatic necrosis (IPN)

Univariate logistic regression analysis (Table 3) was performed to evaluate the predictive power of the absolute lymphocyte count, NLR and other related parameters for IPN. Results indicated significant correlations between IPN and APACHEII score, platelet, CRP, lymphocyte count, NLR, albumin as well as amylase. Further stepwise multivariate logistic regression was constructed and displayed in Table 4. The final model suggested that APACHEII score (Odds Ratio: 1.299, 95 % confidence interval [CI]: 1.153–1.464, *p* < 0.001) and reduced lymphocyte count (Odds Ratio: 0.006, 95 % CI: 1.153–1.464, *p* < 0.001) were strongly and independently associated with IPN. The area under the ROC curve (Fig. 4) showed the reduced absolute lymphocyte count (0.842, 95 % CI: 0.769–0.914, *p* < 0.001) had a moderate to high accuracy in predicting IPN, higher than APACHEII score (0.819, 95 % CI: 0.722–0.917, *p* < 0.001). The optimal cut-offs from ROC curve was 0.66 × 10^9^/L giving sensitivity of 83.7 % and specificity of 66.7 %.

**Table 3:** Univariate logistic regression analysis for IPN

Elements	Odds ratio	95 % Confidence Interval	*p* Value
Age	1.005	0.977–1.034	0.734
Gender	0.447	0.199–1.006	0.052
BMI	1.105	0.942–1.296	0.218
APACHE II score	1.288	1.168–1.421	<0.001
Hemoglobin	0.991	0.976–1.006	0.221
Hematocrit	0.025	0.000–5.185	0.175
Platelet	0.988	0.981–0.996	0.002
CRP	1.010	1.004–1.017	0.001
WBC	0.941	0.865–1.023	0.153
Lymphocyte count	0.020	0.003–0.123	<0.001
NLR	1.059	1.015–1.104	0.008
Albumin	0.858	0.762–0.967	0.012
Amylase	1.001	1.000–1.001	0.034
Lipase	1.000	1.000–1.000	0.750
HLA-DR	0.909	0.806–1.025	0.120
CD3+ T cell	0.945	0.872–1.025	0.173
CD4+ T cell	0.944	0.844–1.055	0.310
CD8+ T cell	0.930	0.807–1.071	0.312
CD4+/CD8+ T cell	0.953	0.254–3.580	0.944

*IPN* infected pancreatic necrosis, *BMI* Body Mass Index, *APACHE II score* Acute Physiology and Chronic Health Enquiry II score, *CRP* C-reactive protein, *WBC* white blood cells, *NLR* Neutrophil-lymphocyte ratio, *ERCP* Endoscopic Retrograde Cholangiopancreatography, *HLA-DR* human leukocyte antigen-DR

**Table 4:** Multivariate stepwise logistical regression and receiver operator characteristic (ROC) curve to predict IPN

Element	Odds Ratio (95 % CI)	*p* Value	AUROC (95 % CI)	*p* Value
Lymphocyte count	0.006 (0.000–0.100)	<0.001	0.842 (0.769–0.914)	<0.001
APACHE II score	1.299 (1.153–1.464)	<0.001	0.819 (0.722–0.917)	<0.001

*ROC* receiver operator characteristic, *IPN* infected pancreatic necrosis, *AUROC* area under receiver operating characteristic curve, *CI* confidence interval, *APACHE II score* Acute Physiology and Chronic Health Enquiry II score

## Discussion

About one third of the patients with necrotizing pancreatitis would develop IPN progressively [14]. IPN would prolong the hospital stay and increase the incidence of complications as well as mortality. Briefly, the development of IPN determines the management of acute pancreatitis and has a great influence on the prognosis. In accordance with previous studies, the incidence of complications and mortality were much higher in the patients with IPN than those without. Also, MOF caused by pancreatic infection or sepsis was the major death cause in both two groups. Hence, it is urgent to find a simple and early marker that could predict IPN at the very onset of the disease. In the literature, several biochemical parameters such as procalcitonin, CRP and NLR have been investigated but turned out with unsatisfying results. Our study first assessed the predictive power of the absolute lymphocyte count for IPN in the patients with acute pancreatitis and demonstrated that the absolute lymphocyte count was a strong predictor for IPN in AP patients with a moderate to high accuracy. Patients who developed IPN in the late course of AP had significantly lower lymphocyte count in the peripheral blood at the initial stage (within 48 h of AP onset) than those without IPN. Contrast to the study of Azab et al., our study indicated that NLR did not show good prognostic value when compared with lymphocyte count [15]. However, in Azab et al.’s study, the primary outcome was severity instead of secondary infection, which might contribute to the difference of the results in the two studies.

Currently, immunosuppression is well accepted as an important risk factor for IPN in AP patients [8]. HLA-DR is a crucial immunological index and shows close relationship with sepsis and late mortality in SAP patients in many studies [16–18]. Early alteration of T lymphocyte subsets is also proved to have significant influence on the prognosis of AP patients [19]. Nonetheless, those immunological indexes need to be examined by flow cytometry, which are not routinely performed in every hospital due to its high cost and complexity. Furthermore, the accuracy of flow cytometer largely depends on the laboratory technician who carried out the experiment.

The absolute lymphocyte count, as a simple immunological index, can roughly and rapidly reflect the general change of the immune system. More importantly, the lymphocyte count can be easily assessed and is available in every hospital. Christophi et al. first evaluated the prognostic value of the absolute lymphocyte count in 104 male patients and 50 female patients with acute pancreatitis. They classified the patients according to their severities, mild and severe group. They found that the mean absolute lymphocyte count in the patients with SAP was significantly lower than those with mild AP and concluded that the absolute lymphocyte count had an accurate predictive power for AP severity. However, major flaws in that study were as follows: First, the patients included in that study were not restricted to those in the acute phase. Second, the results of the statistic analysis were not powerful enough to get such a solid conclusion. More recently, a study performed by Conlledo et al. confirmed that early lymphocyte count was independently associated with increased mortality in patients with sepsis or septic shock [20]. Besides, the study also indicated that the absolute lymphocyte count was closely associated with late infection.

One latest retrospective study by Zeng et al. identified the independent risk factors for pancreatic infection in 163 patients with acute pancreatitis [11]. They performed multiple logistic analyses and concluded that increased lactate dehydrogenase (LDH), high CT severity index, delayed fluid resuscitation and hypoxemia were independent risk factors for predicting IPN in patients with SAP. Nonetheless, they did not integrate the absolute lymphocyte count into their analysis.

Circulating lymphocyte subsets in acute pancreatitis have also been studied for a long time in the literature [21]. Many studies suggested that dysregulation of T lymphocytes played a vital role in the process of acute pancreatitis [22, 23] and down regulation of HLA-DR indicated IPN development and poor prognosis [16, 24]. In our patients, significantly down-regulated HLA-DR was also seen in those AP patients with IPN. However, the relationship between HLA-DR and IPN was not strong enough, probably attributing to the limited sample size. Similar to previous studies [19], the percentage of mature T lymphocyte was also a bit lower in the patients with IPN, as well as the percentages of CD 4+ and CD8+ T lymphocytes. These results demonstrated that the occurrence of immunosuppression at the early stage of acute pancreatitis might be a strong independent risk factor for IPN development in the late stage. To verify this hypothesis, further larger studies might be needed.

Several limitations of our study were listed as follows: Firstly, because of the small number of the IPN patients, the predictive power might be slightly influenced. Secondly, the retrospective nature of the study limited the extension of the study. Lastly, there were some data missing in the respect of HLA-DR and T lymphocyte subsets, which may also have some influence over the results. However, the majority of the abovementioned limitations were not thought to have too much influence on the results as they were equally existed in the two groups.

## Conclusion

In summary, early immunosuppression always occurs in AP patients who might develop IPN in the late stage of the disease. Reduced lymphocyte count at the initial stage of the disease (within 48 h of AP onset), which simply and generally reflects the dysfunction of the immune system, might be an early and powerful predictor for IPN in AP patients.
